# Retroperitoneal Ganglioneuroma: Mimicking an ovarian mass in a child

**DOI:** 10.12669/pjms.313.5980

**Published:** 2015

**Authors:** Hayrünisa Kahraman Esen, Osman Esen, Cesim Irsi

**Affiliations:** 1 Dr. Hayrünisa Kahraman Esen, Department of Pediatric Surgery, Fatih Sultan Mehmet Training and Research Hospital, Istanbul, Turkey; 2 Dr. Osman Esen, Department of Pediatric Surgery, Fatih Sultan Mehmet Training and Research Hospital, Istanbul, Turkey. Department of Anesthesiology and Reanimation, Kocaeli Derince Training and Research Hospital, Kocaeli, Turkey; 3 Dr. Cesim Irsi, Department of Pediatric Surgery, Fatih Sultan Mehmet Training and Research Hospital, Istanbul, Turkey

**Keywords:** Ganglioneuroma, Retroperitoneum, Sympathetic nervous system

## Abstract

Ganglioneuroma are rare, benign neural tumor of sympathetic nervous system originating from neural crest sympathogonia. Pelvic ganglioneuromas may mimic ovarian masses. We present a patient operated with the diagnosis of ovarian tumor which had a pelvic gangliuoneuroma.

**Case:**

An eleven-year old female patient complaining of abdominal pain and a mass was seen at our hospital. On examination a 10×10 cm mass was palpable in the pelvis. Laboratory parameters were all normal and the tumor markers such as β-HCG, AFP, CEA, serum catecholamines were negative. Abdominal ultrasonography and computed tomography showed an 11×6×9 cm solid mass containing calcification. The preoperative diagnosis was an adnexal mass of ovary. The patient was operated under general anesthesia and we found a retroperitoneal mass attached to the spine at L5. The tumoral mass was completely excised. Histopathological examination of tumor was reported as ganglioneuroma. The patient was discharged on seventh day of hospitalization with no neurological deficit.

Retroperitoneal ganglioneuromas are usually present with local mass a benign tumoral course. The preoperative diagnosis may be difficult in pelvic ganglioneuromas due to close similarity with the ovarian masses. The treatment of the ganglioneuroma is total surgical excision and histology provides a confirmatory diagnosis.

## INTRODUCTION

Ganglioneuromas (GNs) are well differentiated; benign, central and peripheral autonomic neural system related tumours mostly originated from sympathetic nervous system.^1^

The most common localization of ganglioneuroma is posterior mediastinum and retroperitoneum. Girls are more commonly affected than boys and generally they are diagnosed under the age of 10 years.^2^ They are hormonally inactive and due to their slow growth pattern, the diagnoses of the ganglioneuromas are made late. The main symptoms of the tumour are abdominal mass and pain because of mass and pressure effect of tumours to adjacent organs.^3^ There is no specific radiological sign for ganglioneuromas and that is why tumours differentiation with other tumours are difficult and the diagnosis are made only by histological examination postoperatively.^4^,^5^

Here in, we report a case of a patient who was preoperatively diagnosed as ovarian tumour and uneventfully operated but she had a diagnosis of ganlioneuroma after histopathological evaluation.

## CASE REPORT

**Fig.1:**
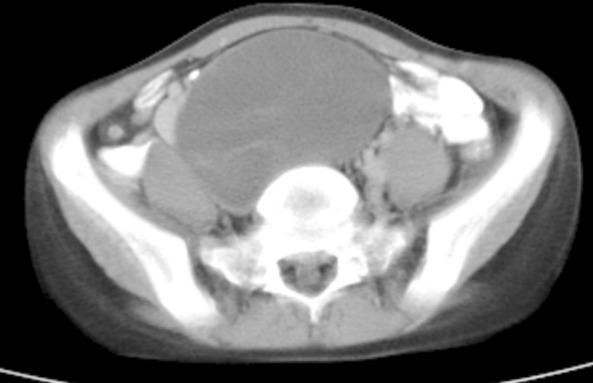
Computed tomography scan shows a mass arising in the retro peritoneum.

An 11-year- old female patient complaining with abdominal pain and abdominal mass was admitted to our hospital. The patient had been suffering from these symptoms for three months and before admission she was diagnosed as abdominal hydatid cyst. On physical examination: a 10×10 cm mass was palpable in the pelvis extending up to the umbilicus and was dull on percussion. Biochemical, haematological, hormonal laboratory parameters were all normal and the tumour markers such as β-HCG, AFP, CEA, serum catecholamines were negative. Serology for hydatid disease was also negative. Abdominal computed tomography showed a 110×63×90 mm mass with solid internal structure, which displaced the right common iliac artery and vein anteriorly. Hyper vascular septa with highly resistant arterialize was observed in the mass (Fig.1).

**Fig.2:**
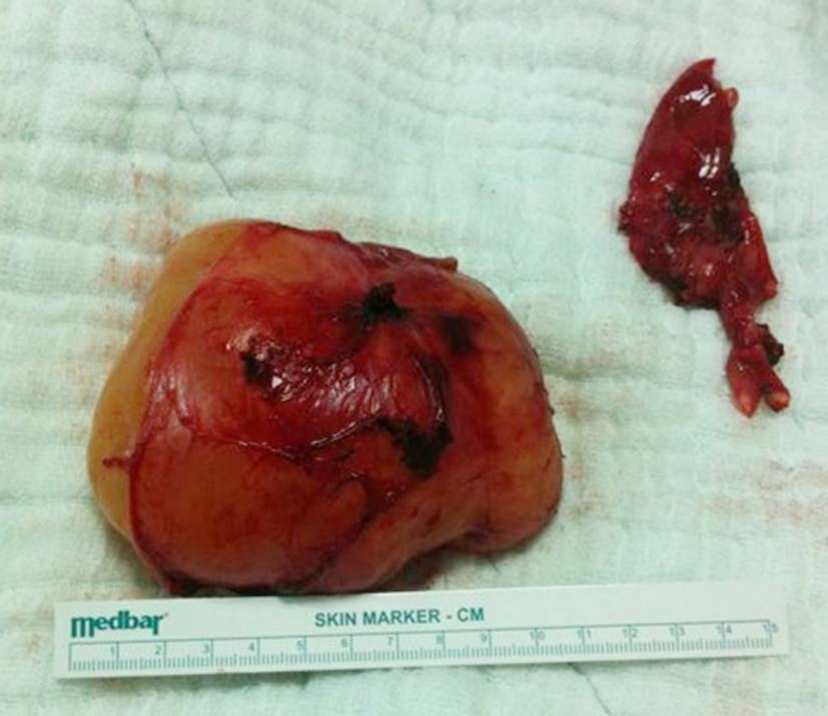
Postoperative photograph of ganglioneuroma and capsule.

The patient was operated under general anaesthesia after preoperative preparations. Although the preoperative diagnosis was an adnexal mass of ovary, intraoperatively it was seen that the mass was retroperitoneally situated. The patient mass was located retroperitoneally at the conjoined with right and left common iliac artery and vein and attached to the L5-S1 vertebrae by posterior capsule. At the posterior of L5 spinal capsule there was a neural connection with the tumour wall. We removed the tumoural mass completely and dissected neural connection which was the main and only connection of spinal cord with the tumour. The excised tumour was well-demarcated, non-cystic and measuring (110×63×90mm (Fig.2).

At the histopathological examination; lobular mass with dirty white appearance in cross section was found. Staining with S-100 dye was reported as ganglioneuroma with schwann type stromal mature ganglion cells and immune positive. The patient’s postoperative neurological examination was normal and was discharged on seventh day of hospitalization.

## DISCUSSION

Neuroblastic tumours from sympathetogonia are divided in to three groups’ as neuroblastomas, ganglioneuroblastomas and ganglioneuromas according to the degree of cellular & extracellular maturation. Ganglioneuraoma are composed of gangliocytes and mature stroma and are benign in nature. Neuroblastoma are the most immature, poorly differentiated and malignant tumours of all the three.^6^,^7^

Ganglioneuroma are mostly seen during infancy and preschool period. The age of diagnosis is generally under 10 years and average age is 7. Girls are three times more affected than boys. The incidence of the tumour is reported to be 1/100.000.^1^,^2^,^6^ GNs are located most commonly in the thoracic cavity (posterior mediastinum) (60-80%), and less commonly in the abdominal cavity (adrenal gland, pelvic, retro peritoneum, sacral and coccygeal sympathetic ganglia, and the organ of Zuckerkandl) (10-15%) or in the cervical region (5%).^2^

They are clinically asymptomatic and, non-functional. Because of their slow growth pattern, diagnosis is usually made at late adolescence age. The diagnosis is made by tumours mass and pressure affect to surrounding tissues and organs. If the tumour grows retroperitoneally, the main symptoms are abdominal pain and distention.^3^,^4^,^8^ In our case, chronic abdominal pain and distension were the presenting symptoms of the patient because of large mass.^6^ GNs generally are radiological well localized, round or oval in shape, lobulated contour, and solid masses with some cases containing partial calcifications.^6^,^9^,^10^ Radiologic imaging such as abdominal U/S, CT & MRI can help in evaluating the size of the mass, it’s composition and it’s relation to adjacent structures. It may be difficult to assess true nature of large pelvic masses by radiology alone and tumour markers like alfaFeto protein, β HCG, and serum catecholamines may help in some cases by the raised tumor markers. It may still be difficult to diagnosis the nature of tumours preoperatively in some cases and definite diagnosis are made postoperatively by histopathological evaluation as in our patient.^5^ In our case, the preoperative diagnosis was an abdominal hydatid cyst or an ovarian mass (disgerminoma). The definite diagnoses were made after resection as neuroganglioma by histopathological examination. Also, a patient reported by Adam et al.^6^ was diagnosed preoperatively as abdominal hydatid cyst had a retroperitoneal ganglioneuroma after resection and histopathological examination.

The main differentiation criterion for neurological tumours was histopathological existence of mature ganglion cell. Ganglioneuroma contains; schwans cells, ganglion cell, fibroses tissue and neural fibers. There are no immature cells, atypical, mitotic structure or necrosis in ganglioneuromas as neurobastomas.^6^

The definite diagnosis of ganglioneuroma in our case was made by existence of: both bundle forming, oval nucleated and spindle shape schwans cells with the ganglion cell appearing as granulated eosinophilic cytoplasm. Surgically total resection of the tumour is the main treatment and there is no recurrence reported.^6^ The tumour was totally removed surgically in our case.

In conclusion, the preoperative diagnosis of retroperitoneal ganglioneuromas due to radiological confusion with other tumours are very difficult. We should keep in mind that retroperitoneal masses may be a ganglioneuroma and the diagnosis can be made only by histopathological evaluation postoperatively.
